# Editorial: Classification of foods according to their processing level

**DOI:** 10.3389/fnut.2023.1128477

**Published:** 2023-02-06

**Authors:** Paulo José do Amaral Sobral, Andrzej Lenart, Marco Dalla Rosa

**Affiliations:** ^1^Department of Food Engineering, Faculty of Animal Science and Food Engineering, University of São Paulo, Pirassununga, Brazil; ^2^Department of Food Engineering and Process Management, Faculty of Food Sciences, Warsaw University of Life Sciences, Warsaw, Poland; ^3^Department of Agricultural and Food Sciences, Alma Mater Studiorum-Università di Bologna, Bologna, Italy

**Keywords:** food classification, minimally processed food, processed food, ultra-processed food, unity operations, food technology

Much of the progress of the food industry can be linked to advances in Food Science, Technology, and Engineering (FSTE) since the period following WWII (1, 2). Today, consumers have access to a greater diversity of food than 70 years ago; however, even though food is now more nutritious and safe, some health problems related to eating habits (3) have been described in the media and scientific journals. Obesity and cardiovascular diseases, among others, have been associated with the high consumption of salt, sugar, and other ingredients of industrialized foods. This topic was discussed by Amorim et al. in a review that is a systemic and fundamental overview that also examines hunger, food loss and waste, and sustainability.

Recently, Food Guidelines (FG) have been created in several countries to avoid or mitigate these health problems linked to human nutrition. In particular, some of these FGs are based on concepts and classifications of food with an emphasis on the NOVA classification, in which foods have been classified into four types (4): (1) unprocessed and minimally processed foods (MPF), (2) processed culinary ingredients (PCI), (3) processed foods (PF), and (4) “ultra-processed” foods (UPF). Notably, the authors of this classification consider that UPF are not “real food” (4).

Among these classifications, the only new classification that NOVA created was the UPF category. The term “minimally processed foods” (MPF) had already been used in the FSTE literature for decades (5), so only the UPF was a new term. Although this new food classification was based on amounts/varieties of food ingredients added to the formulation, it did not necessarily focus on the important role of unit processing operations. For FSTE professionals, food processing entails submitting raw material to a sequence of processing steps known as unit operations. In this regard, a paper submitted by Tadini and Gut overviewed the mainstream heating unit operations used in the food industry. These authors highlighted the benefits of these unit operations to process raw materials into palatable and safe foodstuff for consumption. Their findings demonstrated that there are no drastic or catastrophic unit operations for the so-called “ultra-processed” foods.

Furthermore, some controversial opinions regarding the classified food themselves can be noted within the FG classifications. For example, spices were considered PF, but they must be classified as being PCI because they are not usually consumed on their own. Also, cheeses were identified as UPF by NOVA, but many kinds of cheese are produced only with milk, salt, and ferment, i.e., a minimal amount of ingredients. Moreover, the NOVA classification system classified chocolate in a generalized manner as an UPF, but dark chocolate is usually produced only with cocoa, cocoa butter, and a little sugar. Thus, it would not classify as an UPF.

Other ambiguities can also be observed in other NOVA classifications. For example, natural yogurt was classified as a PF, while fruit yogurt was classified as an UPF, although both foodstuffs are processed with the same unit operations. Evidently, these points can create consumer confusion (6). Concerns about these conceptual questions were raised in a paper published by Sadler et al., who stated their misgivings about NOVA's classifications as follows: (1) broad concepts that need differentiation, (2) disagreements on scope and degree of processing, (3) the role of food processing within the food system and the dilemmas in identifying risks and benefits, and (4) the challenges of different perspectives and interests for risk communication, identifying a need for further interdisciplinary dialogue, including public engagement. Similar concerns and conclusions were described in another recent paper (7).

It is evident that these food classifications have good intentions concerning the health of consumers; nevertheless, the quality and the role of food processing in the evolution of humanity and its contribution to the assurance of food security on a global scale must be recognized (1, 5, 6). For this reason, the principles, ambiguities, generalizations, and mistaken concepts should be critically examined and clarified in the light of the body of scientific knowledge as considered by FSTE. Furthermore, Rego published an opinion paper regarding this subject and observed that harmonizing concepts and interests that are quite distinct and prone to antagonism is not a simple task. This author stated that “food system leaders must have full authority to name a mediator agent to this process, a role suggested to regulatory authorities.”

The authority to classify foods based on their level of processing (or amount of ingredients) has many limitations. For example, as discussed by Jideani et al. in a review about African food processing, most plant foods from sub-Saharan Africa are still minimally prepared in their natural state, and some low-income countries in Africa suffer with unreliable road networks, poor storage infrastructures, and electricity issues, which favors food spoilage. Thus, it is in these countries that food processing on an industrial basis must be encouraged to guarantee food shelf life. Given this, these authors proposed a discussion on another food classification system that also considered “scoring foods in a hierarchy where a holistic index is first applied.” This demonstrates the complexities of determining food processing classifications.

In summary, when reviewing papers included in this Research Topic, readers should try to understand the actual relevance and full scope of food classifications. Certainly, readers should also realize that the existence of these types of classifications encourages FSTE professionals and manufacturers to develop low-processed foods, reduce additives, and implement transparency and clean label approaches in general.

## Author contributions

All authors listed have made a substantial, direct, and intellectual contribution to the work and approved it for publication.
